# Role of Nutritional Status in Acute Coronary Syndrome Patients with Diabetes

**DOI:** 10.3390/medicina61040740

**Published:** 2025-04-17

**Authors:** Özlem Seçen, Muhammed Fuad Uslu

**Affiliations:** 1 Department of Cardiology, Elazığ Fethi Sekin City Hospital, 23280 Elazığ, Turkey; 2 Department of Internal Medicine, Elazığ Fethi Sekin City Hospital, 23280 Elazığ, Turkey; dr.fuslu@gmail.com

**Keywords:** acute coronary syndrome, STEMI, NSTEMI, prognostic nutrition index

## Abstract

*Background and Objectives:* This study aims to investigate the effect of Type 2 diabetes mellitus (DM) on nutritional status in acute coronary syndrome (ACS) patients and its relationship with various metabolic and hematologic parameters. *Materials and Methods:* A retrospective and cross-sectional design was used to analyze 485 acute coronary syndrome (ACS) patients who underwent angiography at Fethi Sekin City Hospital between 1 January 2020 and 1 January 2025. Clinical data, biochemical parameters (hemogram, glucose, creatinine, uric acid, lactate dehydrogenase (LDH), albumin, and cholesterol levels) were retrospectively analyzed. The Prognostic Nutrition Index (PNI) and CONUT score were calculated manually. *Results*: A total of 485 patients were included in this study. Patients were divided into two groups: patients with DM (n = 167) and patients without DM (n = 318). Glucose levels (*p* < 0.001) and triglyceride levels (*p* = 0.014) were significantly higher in patients with diabetes, while LDL cholesterol and total cholesterol levels were lower (*p* < 0.01). In addition, hemoglobin (*p* < 0.001), albumin (*p* = 0.010), and PNI scores (*p* = 0.014) were lower in patients with diabetes. Although CONUT scores were higher in patients with diabetes, this difference was not statistically significant (*p* = 0.267). Significant differences were observed in lipid profile and inflammation parameters in STEMI and NSTEMI subgroups, especially in patients with diabetes. In particular, triglyceride and neutrophil levels were found to be higher in NSTEMI patients among patients with diabetes. *Conclusions:* The PNI score may be a useful prognostic tool for predicting cardiovascular complications and determining treatment strategies in acute coronary syndrome patients with diabetes mellitus in whom nutritional status, inflammation, and lipid metabolism are significantly correlated.

## 1. Introduction

Cardiovascular disease (CVD) is one of the leading causes of mortality worldwide and represents a significant public health concern. Acute coronary syndrome (ACS) is characterized by a sudden reduction in blood flow to the heart, resulting in myocardial ischemia. The primary subtypes of ACS include unstable angina, non-ST-elevation myocardial infarction (NSTEMI), and ST-elevation myocardial infarction (STEMI) [1,2,3]. Patients with diabetes are at an increased risk of developing CVD. As one of the major macrovascular complications in diabetic patients, CVD accounts for 50.3% of all deaths, with coronary artery disease (CAD) being the primary contributor, responsible for 29.7% of cases [4].

Standard treatment approaches for ACS include thrombolytic therapy and primary percutaneous coronary intervention (pPCI) to achieve early reperfusion. Although these treatments reduce the incidence of clinical events, patients remain at risk for recurrent adverse cardiovascular events [5,6,7]. The development of atherosclerosis and plaque formation within the subintimal layers of the coronary arteries are influenced by lipid profiles, making this process a predictor of acute myocardial infarction (AMI) [8,9,10]. In dyslipidemia, elevated levels of total cholesterol (TC), triglycerides (TGs), and low-density lipoprotein cholesterol (LDL-C), along with decreased levels of high-density lipoprotein cholesterol (HDL-C), significantly increase the risk of myocardial infarction (MI) [11,12,13].

Several recent studies have demonstrated that malnutrition is associated with increased in-hospital mortality, long-term mortality, and adverse cardiovascular events in patients with acute coronary syndrome (ACS), acute myocardial infarction (AMI), acute heart failure (HF), chronic heart failure, and atrial fibrillation (AF) [14,15,16,17,18,19,20]. Nutrition is one of the key modifiable risk factors for cardiovascular health in individuals with or without diabetes [21,22]. In particular, malnutrition is an important and common comorbidity in diabetic patients and is associated with in-hospital mortality and long-term outcomes. There is also evidence of worsening clinical outcomes, especially in geriatric patients, where diabetes is associated with poor nutritional status [23,24]. Diabetes is known to be associated with increased systemic inflammation. A high degree of malnutrition is associated with high levels of inflammation, which translates into increased atherosclerotic burden. The relationship between all these has recently been described as the malnutrition–inflammation–atherosclerosis syndrome [25].

Quantifying nutritional status has always been challenging, and several tools have been proposed in the literature to classify patients as malnourished [26]. One such tool is the Prognostic Nutritional Index (PNI), which provides a quantitative assessment of a patient’s nutritional and immune status. It is calculated based on serum albumin levels and total lymphocyte count [27].

Another widely used tool for assessing nutritional status is the Controlling Nutritional Status (CONUT) score. The CONUT score is a simple and accessible measure derived from serum albumin, total cholesterol, and lymphocyte count, serving as a comprehensive marker of malnutrition. Previous studies have demonstrated that a higher CONUT score is associated with worse outcomes in patients with cardiovascular disease, including coronary artery disease and previous myocardial infarction [28,29,30].

In this study, we aimed to assess the impact of nutritional status in patients with acute coronary syndrome (ACS), with a particular focus on its interaction with diabetes mellitus. We also sought to explore the potential role of malnutrition in contributing to adverse clinical outcomes in this population in the context of the emerging malnutrition–atherosclerosis association.

## 2. Materials and Methods

### 2.1. Participants and Study Design

This study was approved by the Fethi Sekin City Hospital Non-Interventional Local Ethics Committee with project number 2025/2-2 (Meeting Date: 16 January 2025) and was conducted in accordance with the ethical principles outlined in the Declaration of Helsinki. The study has a retrospective and cross-sectional design and includes patients diagnosed with acute coronary syndrome (ACS) who underwent angiography at Elazığ Fethi Sekin City Hospital between 1 January 2020 and 1 January 2025.

**Figure 1:**
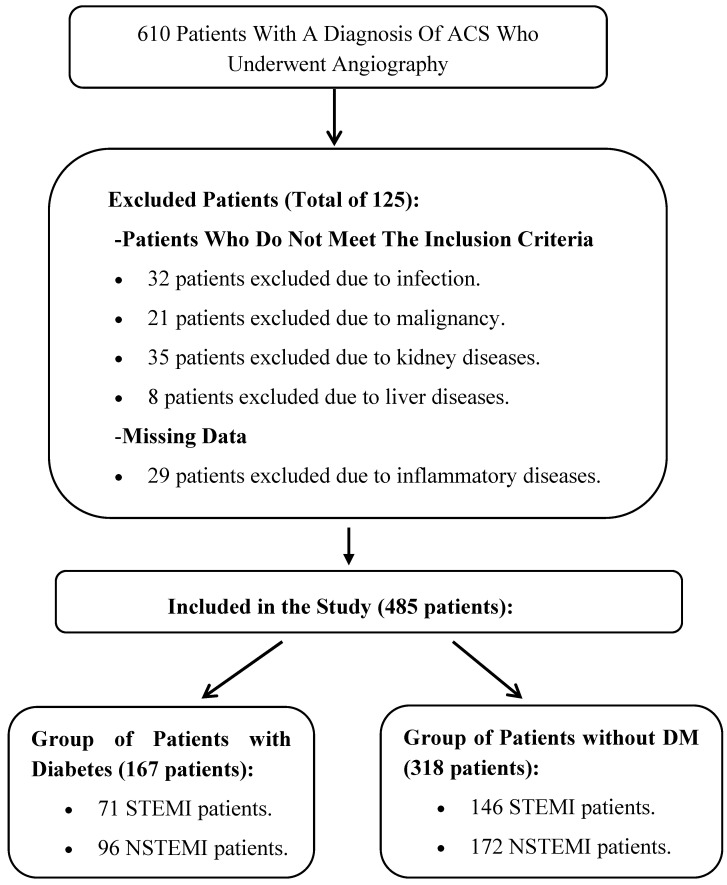
The flowchart of participant selection.

The patients were classified into two groups: patients with DM ACS and patients without DM ACS. Patient records from the hospital database were reviewed, and retrospective analyses were conducted on the blood test results, including hemogram, biochemistry, and cholesterol levels, of patients who underwent angiography. Patients aged 18 years and older without a history of active infection, malignancy, inflammatory disease, renal disease, or liver disease were included in this study. After excluding 125 patients who did not meet the inclusion criteria and those with missing data, a total of 485 patients were included in the final analysis (Figure 1).

### 2.2. Methods of Data Collection

To record the data used in this study, the authors developed a sociodemographic and clinical data form based on clinical experience and the existing literature. This form included demographic information such as age and gender, as well as findings from electrocardiography (ECG), echocardiography (ECHO), and angiography, which were obtained from patient records.

In addition, laboratory parameters including glucose, creatinine, uric acid, lactate dehydrogenase (LDH), albumin, white blood cell (WBC) count, hemoglobin, neutrophil count, lymphocyte count, platelet count, triglycerides (TGs), high-density lipoprotein (HDL), and low-density lipoprotein (LDL) were recorded from the patients’ test results obtained during hospitalization. Based on these data, the following parameters were calculated:

Prognostic Nutritional Index (PNI): Albumin value (g/L) + (5 × lymphocyte count [10^9^/L]) [31].

Controlling Nutritional Status (CONUT) score: This is used to assess nutritional risk, calculated based on serum albumin concentration, total cholesterol level, and total lymphocyte count, as previously described. Each parameter is assigned a specific score. If the serum albumin level is above 3.5 g/dL, it receives 0 points; between 3.0 and 3.5 g/dL, it receives 1 point; between 2.5 and 2.9 g/dL, it receives 2 points; and below 2.5 g/dL, it receives 3 points. If the total cholesterol level is above 200 mg/dL, it receives 0 points; between 150 and 200 mg/dL, it receives 1 point; between 100 and 149 mg/dL, it receives 2 points; and below 100 mg/dL, it receives 3 points. For the total lymphocyte count, if it is above 1500 cells/µL, it receives 0 points; between 1200 and 1500 cells/µL, it receives 1 point; between 800 and 1199 cells/µL, it receives 2 points; and below 800 cells/µL, it receives 3 points. The sum of these three parameters provides information regarding the nutritional status. According to the CONUT score, 0–4 points indicate normal nutritional status, 5–8 points indicate moderate malnutrition or risk, and 9–12 points indicate severe malnutrition or high risk [28].

Blood samples were collected from the patients at the time of admission and examined on the same day at the central laboratory using routine laboratory techniques. In our hospital, hemogram analyses were performed using a DXH-800 device (Beckman Coulter, Inc., Miami, FL, USA), and biochemical parameters were analyzed with a Beckman AU-5800 device (Beckman Coulter Diagnostics, Indianapolis, IN, USA). Based on these parameters, PNI and CONUT scores were calculated manually.

### 2.3. Statistical Analysis

Statistical analyses were performed with SPSS v. 22 (Statistical Package for the Social Sciences; SPSS Inc., Chicago, IL, USA). The normality of distribution of continuous variables was determined using the Kolmogorov–Smirnov test. A one-way ANOVA was used for the comparison of more than two groups. A Post Hoc Tukey test was used for pairwise group comparisons after the one-way ANOVA. The chi-square test or Fisher exact test was used to analyze categorical data. Numerical data were expressed as mean ± SD and categorical data as percentage (%). Logistic regression analysis was used to calculate risk factors, starting with a univariate analysis, followed by a multivariate analysis for variables found significant in the first step. The statistical significance level was accepted as *p* < 0.05.

## 3. Results

A total of 485 patients were included in this study and categorized into two groups: patients with DM (n =167) and patients without DM (n = 318). There was no significant difference in age between the groups (patients with DM: 65.72 ± 11.10 years vs. patients without DM: 64.12 ± 13.18 years, *p* = 0.234) (Table 1). However, statistically significant differences were observed in certain biochemical and hematologic parameters.

Glucose levels were significantly higher in the DM group compared to the group of patients without DM (186.11 ± 95.71 mg/dL vs. 129.54 ± 49.23 mg/dL, *p* < 0.001), which aligns with the metabolic nature of DM. Furthermore, triglyceride levels were significantly elevated in the DM group (167.74 ± 160.57 mg/dL vs. 134.88 ± 75.93 mg/dL, *p* = 0.014), suggesting an increased risk of dyslipidemia among patients with DM. In contrast, low-density lipoprotein (LDL) cholesterol and total cholesterol levels were lower in patients with DM (99.65 ± 37.09 mg/dL vs. 111.31 ± 40.83 mg/dL, *p* = 0.001 and 164.95 ± 45.53 mg/dL vs. 177.22 ± 49.52 mg/dL, *p* = 0.008). This may be associated with more frequent lipid-lowering treatments in patients with DM (Table 1).

Another important finding was that albumin levels were significantly lower in the group of patients with DM (36.63 ± 4.77 g/L vs. 37.84 ± 5.11 g/L, *p* = 0.010). This may indicate an increased risk of inflammation or malnutrition in patients with DM. Hemoglobin levels were also significantly lower in the group of patients with DM (13.36 ± 2.00 g/dL vs. 14.15 ± 1.98 g/dL, *p* < 0.001), suggesting a possible association between DM and anemia (Table 1).

The Prognostic Nutritional Index (PNI), which reflects nutritional and inflammatory status, was also significantly lower in the group of patients with DM (51.09 ± 18.93 vs. 53.92 ± 18.88, *p* = 0.014), suggesting that nutritional status may be compromised in patients with DM (Table 1).

When comparing the subgroups of ST-segment elevation myocardial infarction (STEMI) and non-ST-elevation myocardial infarction (NSTEMI), triglyceride levels were significantly higher in patients with DM with NSTEMI compared to those in the STEMI group (189.66 ± 190.87 mg/dL vs. 138.30 ± 101.35 mg/dL, *p* = 0.007). This finding suggests that lipid metabolism may be associated with the different subtypes of myocardial infarction (MI) (Table 2).

Additionally, hemoglobin levels were significantly higher in the group of patients without DM compared to the DM group among STEMI patients (14.42 ± 1.96 g/dL vs. 13.91 ± 1.98 g/dL, *p* = 0.022), indicating that anemia may be more prevalent in patients with DM. Furthermore, platelet (PLT) levels in STEMI patients were lower in patients without DM compared to the DM group (234.07 ± 66.51 vs. 250.09 ± 68.61, *p* = 0.019) (Table 2).

Another notable finding was that neutrophil levels were significantly higher in DM patients in the NSTEMI group compared to those in the STEMI group (12.84 ± 33.93 vs. 6.87 ± 3.75, *p* = 0.016). This suggests that the inflammatory response may be more pronounced in patients with DM and with NSTEMI (Table 2).

In the binary logistic regression analysis conducted to identify factors associated with mortality (exitus), the model was found to be statistically significant (χ^2^(2) = 50.95, *p* < 0.001). According to the results, age (B = 0.009, *p* = 0.041), glucose level (B = 0.009, *p* = 0.001), and glomerular filtration rate (GFR) (B = −0.041, *p* = 0.001) were significantly associated with the risk of exitus. An increase in age and glucose level was associated with a 7.6% (OR = 1.076) and 0.9% (OR = 1.009) higher risk of exitus, respectively, while an increase in GFR was associated with a 4% reduction in the risk (OR = 0.960). Platelet count showed a borderline statistical significance (B = 0.007, *p* = 0.064). The explanatory power of the model was 23.7% based on the Cox–Snell R^2^ and 34.7% based on the Nagelkerke R^2^ (Table 3).

In conclusion, the findings presented in Table 1 indicate that patients with DM exhibit a distinct metabolic and inflammatory profile, characterized by elevated glucose and triglyceride levels, as well as lower levels of low-density lipoprotein (LDL) and total cholesterol. Furthermore, reduced hemoglobin and albumin levels, along with lower Prognostic Nutritional Index (PNI) scores, suggest that both nutritional status and hematologic parameters are adversely affected in patients with DM.

The results in Table 2 reveal significant differences in lipid profiles, inflammation markers, and hematologic parameters among the STEMI and NSTEMI subgroups, particularly in patients with DM. Notably, patients with DM with NSTEMI had higher triglyceride and neutrophil levels, suggesting that inflammatory processes may be more pronounced in this subgroup. In STEMI patients, higher hemoglobin levels and lower platelet counts in the patients without DM group imply that hematologic changes may be associated with cardiovascular risk.

## 4. Discussion

This study was based on scores such as the Controlling Nutritional Status (CONUT) score and the Prognostic Nutritional Index (PNI), which assess nutritional status. The findings revealed the negative effects of DM on nutritional status, although the difference in the CONUT score was not statistically significant. This suggests that DM may be associated with inflammation and nutritional disorders, indicating that the nutritional status of patients with DM may be compromised. The decrease in PNI scores highlights the importance of nutritional monitoring in the clinical management of patients, as these parameters may help predict the risks of complications in individuals with more advanced stages of DM. Furthermore, this study demonstrates that these scores can serve as potential assessment tools in the management of DM. The fact that this study was conducted in patients with DM is a significant factor that enhances its value. DM is a high-risk condition for metabolic and cardiovascular diseases, and parameters such as nutritional status, inflammation levels, and lipid profiles play a critical role in understanding the overall disease status and risk of complications in these patients.

In recent years, malnutrition has garnered widespread attention as a prognostic indicator in various diseases [32,33,34]. Several tools have been developed to quantify nutritional status and classify patients as malnourished [26]. One such tool is the Prognostic Nutritional Index (PNI), calculated by summing serum albumin and total lymphocyte counts [27]. A comprehensive evaluation of the relationship between PNI and coronary artery disease (CAD) outcomes in a meta-analysis by Zhang et al. demonstrated that low PNI scores were associated with a 67% increase in mortality and a 57% increase in major adverse cardiovascular events (MACEs) in patients with CAD. The findings of this study align with those of other malnutrition assessment tools used in patients with CAD, suggesting that the PNI can be effectively employed in the risk stratification of these patients [35]. Keskin et al. found that low PNI was associated with a decreased survival rate in the acute coronary syndrome (ACS) group [36]. In the study by Wada et al., patients with low PNI values exhibited reduced survival rates among those with stable coronary artery disease (CAD) [37]. Additionally, Li et al. demonstrated that CAD patients with DM and low PNI (L-PNI) had the highest risk of all-cause mortality; notably, the risk associated with L-PNI outweighed that associated with DM, indicating that DM exacerbates the adverse effects of L-PNI [38]. Considering malnutrition in patients with DM, studies have shown that individuals in this group often experience a negative nitrogen balance due to increased protein catabolism and excretion, coupled with decreased protein anabolism. This situation can create a vicious cycle that heightens the risk of malnutrition, exacerbating insulin resistance and negatively impacting the overall health status of patients. Furthermore, patients with L-PNI and no DM face a higher risk of all-cause mortality compared to those with high PNI and DM, consistent with previous studies suggesting that the mortality risk associated with malnutrition is greater than that related to other chronic comorbidities [23]. Similarly to the findings of other studies, the lower PNI scores identified and emphasized in our study suggest that nutritional status is compromised and hematologic parameters are adversely affected in patients with DM. Low PNI scores may be associated with increased inflammatory processes and alterations in immune system responses, particularly among individuals with DM. Chronic inflammation induced by DM may elevate the risk of malnutrition by impairing nutrient absorption and metabolism. Additionally, decreased lymphocyte counts and low serum albumin levels may indicate a weakened immune system and heightened inflammatory status, which could contribute to increased cardiovascular events and mortality in patients with DM. In conclusion, the assessment of PNI in patients with DM may serve as an important indicator for determining nutritional status and predicting patient prognosis.

As is well known, the Controlling Nutritional Status (CONUT) score evaluates nutritional status by utilizing serum albumin, lymphocyte counts, and total cholesterol values, providing insights into immune status. Studies have demonstrated that a low CONUT score is associated with poorer prognosis and higher mortality across various diseases, exhibiting greater predictive value compared to other malnutrition scoring systems [15,39]. Kalyoncuoglu et al. found that a high CONUT score served as a predictor of major adverse cardiac and cerebrovascular events in elderly patients undergoing percutaneous intervention for non-ST-elevation myocardial infarction (NSTEMI) [39]. Similarly, Boyraz et al. showed that the CONUT score could be utilized to predict the prognosis of patients over 65 years of age with NSTEMI [40]. Additionally, Raposeiras et al. noted that malnutrition is prevalent in patients with acute coronary syndrome and is strongly linked to increased mortality and cardiovascular events [19]. A recent meta-analysis by Lai et al., which included 37,303 ACS patients from 30 studies worldwide, demonstrated that malnutrition was significantly associated with all-cause mortality following ACS [41]. Chen B. et al. reported that high CONUT scores and NLR (neutrophil-to-lymphocyte ratio) values may predict adverse clinical outcomes and mortality in patients with acute myocardial infarction (AMI). Furthermore, CONUT score has been identified as an independent predictor of major adverse cardiac events (MACEs) in AMI patients, and it has been suggested that MACE risk stratification could be improved by a joint analysis of CONUT score and NLR. However, it is also emphasized in the literature that malnutrition may be an indicator of inflammation [42]. However, in our study, no statistically significant difference was found between the groups in terms of CONUT score. This finding may be due to factors such as different demographic and clinical characteristics of our patient population, the retrospective design of this study, and the exclusion of patients with missing data. In addition, the parameters measured in our study may not have enough statistical power to show the prognostic role of the CONUT score. In addition, since inflammatory indices were not evaluated in our study, only limited and indirect information about inflammation could be obtained. Therefore, larger patient cohorts and multicenter, prospective studies are needed to further clarify the clinical significance and prognostic value of the CONUT score.

Trimarchi et al. [43] used the formula BMI × albumin/NLR to calculate the Advanced Lung Cancer Inflammation Index (ALI), which allows for a comprehensive assessment of inflammatory and nutritional status. In their study, in a cohort of ST-elevation myocardial infarction (STEMI) patients undergoing primary percutaneous coronary intervention (pPCI), a cut-off value of 10 was set for ALI, which best predicted all-cause mortality. In particular, patients with an ALI ≤ 10 were shown to have a 2.3-fold increased risk of all-cause mortality compared to those with an ALI > 10. Furthermore, in 217 consecutive patients with acute myocardial infarction complicated by cardiogenic shock, ALI ≤ 12.69 was found to be an independent predictor of 30-day mortality (HR: 3.327; 95% CI: 2.053–5.389; *p* < 0.001) and 30-day major cardiovascular events (MACE) (HR: 2.250; 95% CI: 1.553–3.260; *p* < 0.001). Furthermore, the capacity of ALI to predict all-cause mortality was found to be significantly higher than the commonly studied neutrophil-to-lymphocyte ratio (NLR). In our study, ALI could not be used because body mass index (BMI) data were not available. However, we think that we contributed to a similar hypothesis by using PNI and CONUT scores, which also provide information about nutritional status and inflammation. We believe that more comprehensive and prospective studies to be conducted in the future will make important contributions to the literature like our current study.

This study has several limitations. First and foremost, the retrospective design restricts the results to the available data, making it challenging to draw definitive conclusions about causal relationships. Additionally, the exclusion of patients with missing data may limit the generalizability of the findings. Another limitation is that this study was conducted at a single center, which reduces the validity of the results by preventing the evaluation of patients with diverse demographic and clinical characteristics in a larger sample. The lack of a significant difference in CONUT scores may be attributed to the insufficient sensitivity of the measurements used or the influence of various clinical variables. Moreover, a broader range of biochemical parameters and extended follow-up periods may be necessary to evaluate the nutritional status of patients with DM more comprehensively. Another important limitation of our study is that we could not assess the medications used, BMI data, recent dietary habits, and a more detailed disease history. These factors may significantly impact patients’ nutritional and overall health status. The absence of this information complicates the interpretation of our findings. Factors such as medication use, dietary habits, and disease history are critical for understanding the effects of DM on nutritional status. Future studies could enhance the accuracy of results by collecting and analyzing these data more thoroughly. Lastly, the nutritional assessment scores utilized in this study were limited to a specific time frame, which precluded a dynamic examination of the relationship between patients’ nutritional status and the development of complications. To address these limitations, larger, multicenter prospective studies are needed to robustly validate the findings.

## 5. Conclusions

The results of this study provide significant insights by evaluating the negative effects of DM on nutritional status through CONUT and PNI scores. The findings suggest that DM may be associated with inflammation and nutritional disorders, which can adversely impact the nutritional status of patients. The decrease in PNI scores underscores the importance of nutritional monitoring in predicting the risk of complications in more advanced stages of DM. Our study indicates that CONUT and PNI scores may serve as useful assessment tools in determining the prognosis of patients with DM. However, the lack of a significant difference in the CONUT score may be attributed to demographic and clinical variations, as well as the influence of various patient characteristics. Therefore, we anticipate that the prognostic value of these scores will become more apparent in future larger-scale and multicenter studies.

## Figures and Tables

**Table 1:** Comparison of clinical and biochemical parameters of patients with DM and patients without DM.

GROUPS		Patients with DM	Patients Without DM	*p*
N (F/M)		167 (62/105)	318 (105/253)	<0.001
Age		65.72 ± 11.10	64.12 ± 13.18	0.234
Glucose (mg/dL)		186.11 ± 95.71	129.54 ± 49.23	<0.001
LDH (U/L)		409.90 ± 288.51	415.81 ± 463.00	0.648
Albumin (g/L)		36.63 ± 4.77	37.84 ± 5.11	0.010
Triglyceride (mg/dL)		167.74 ± 160.57	134.88 ± 75.93	0.014
LDL (mg/dL)		99.65 ± 37.09	111.31 ± 40.83	0.001
Cholesterol (mg/dL)		164.95 ± 45.53	177.22 ± 49.52	0.008
HDL (mg/dL)		39.35 ± 10.21	39.81 ± 9.81	0.611
Uric Acid (mg/dL)		5.78 ± 2.02	5.87 ± 2.23	0.697
WBC (10^9^/L)		11.32 ± 3.63	11.46 ± 4.45	0.957
Hemoglobin (g/dL)		13.36 ± 2.00	14.15 ± 1.98	<0.001
Platelet (10^9^/L)		250.74 ± 69.35	241.45 ± 67.86	0.067
Neutrophil (10^9^/L)		10.30 ± 25.95	7.16 ± 4.06	0.192
Lymphocyte (10^9^/L)		2.89 ± 3.10	3.22 ± 3.60	0.348
Creatinine (mg/dL)		1.16 ± 0.99	1.00 ± 0.54	0.118
EF (%)		47.4 ± 11.19	49.73 ± 10.27	0.037
eGFR		73.37 ± 25.59	81.18 ± 23.54	<0.001
Hyperlipidemia	Yes, n (%)	97 (58.1)	152 (47.8)	0.031
	No, n (%)	70 (41.9)	156 (52.2)	
PNİ Score		51.09 ± 18.93	53.92 ± 18.88	0.014
Malnutrition state	Normal, n (%)	137 (82)	292 (91.8)	0.004
	Moderate, n (%)	13 (7.8)	8 (2.5)	
	Severe, n (%)	17 (10.2)	18 (5.7)	
CONUT Score		2.44 ± 2.66	2.02 ± 2.12	0.267
Malnutrition state	Normal, n (%)	86 (51.5)	165 (51.9)	0.038
	Mild, n (%)	45 (26.9)	113 (35.5)	
	Moderate, n (%)	30 (18)	35 (11)	
	Severe, n (%)	6 (3.6)	5 (1.6)	

**N (F/M):** number of patients (female/male); **LDH:** lactate dehydrogenase; **LDL**: low-density lipoprotein cholesterol; **HDL**: high-density lipoprotein cholesterol; **WBC:** white blood cell count; **EF:** ejection fraction; **eGFR:** estimated glomerular filtration rate; **PNI Score:** Prognostic Nutritional Index score; **CONUT Score**: Controlling Nutritional Status score; DM: diabetes mellitus.

**Table 2:** Comparison of STEMI and NSTEMI subgroups in terms of patients with DM and patients without DM.

	Patients with DM			Patients Without DM			P*
MI GROUPS	STEMI	NSTEMI	*p*	STEMI	NSTEMI	*p*	
N (F/M)	71 (29/42)	96 (33/63)	0.392	146 (26/120)	172 (40/132)	0.233	
Age	65.45 ± 11.50	65.92 ± 10.85	0.719	63.12 ± 13.39	64.97 ± 12.98	0.162	0.315
Glucose (mg/dL)	189.87 ± 96.33 ^a^	183.33 ± 95.66 ^a^	0.503	130.21 ± 55.72 ^b^	128.97 ± 43.14 ^b^	0.836	<0.001
LDH (U/L)	422.71 ± 274.44	400.33 ± 299.81	0.437	418.64 ± 354.04	413.66 ± 532.40	0.369	0.986
Albumin (g/L)	36.54 ± 5.49	36.70 ± 4.19	0.675	38.03 ± 4.93	37.67 ± 5.27	0.627	0.078
Triglyceride (mg/dL)	138.30 ± 101.35 ^a^	189.66 ± 190.87 ^b^	0.007	132.08 ± 71.41 ^a^	137.23 ± 79.67 ^a^	0.648	<0.001
LDL (mg/dL)	102.57 ± 38.46 ^a,b^	97.42 ± 36.07 ^a^	0.588	113.45 ± 39.01 ^b,c^	109.50 ± 42.32 ^a,b^	0.342	0.015
Cholesterol (mg/dL)	163.41 ± 48.40	166.09 ± 43.52	0.649	178.53 ± 45.24	176.11 ± 52.99	0.414	0.062
HDL (mg/dL)	40.65 ± 9.06	38.38 ± 10.94	0.120	40.20 ± 9.71	39.49 ± 9.91	0.500	0.431
Uric Acid (mg/dL)	5.78 ± 2.13	5.78 ± 1.95	0.891	6.04 ± 2.55	5.74 ± 1.94	0.527	0.683
WBC (10^9^/L)	11.00 ± 3.97	11.56 ± 3.35	0.140	11.62 ± 3.65	11.33 ± 5.04	0.150	0.744
Hemoglobin (g/dL)	13.50 ± 1.90 ^a,b^	13.26 ± 2.07 ^b^	0.590	14.42 ± 1.96 ^b,c^	13.91 ± 1.98 ^a,c^	0.022	<0.001
Platelet (10^9^/L)	247.66 ± 67.05	253.02 ± 71.26	0.445	250.09 ± 68.61	234.07 ± 66.51	0.019	0.086
Neutrophil (10^9^/L)	6.87 ± 3.75 ^a,b,c^	12.84 ± 33.93 ^a^	0.016	7.03 ± 4.26 ^c^	7.26 ± 3.89 ^b,c^	0.534	0.015
Lymphocyte (109/L)	3.39 ± 4.20	2.52 ± 1.85	0.597	3.48 ± 3.14	3.00 ± 3.94	0.298	0.161
Creatinine (mg/dL)	1.12 ± 1.01	1.18 ± 0.99	0.116	1.00 ± 0.55	1.01 ± 0.53	0.826	0.156
EF (%)	46.06 ± 10.48 ^a^	48.39 ± 11.64 ^a,b^	0.184	48.9 ± 10.52 ^a,b^	50.44 ± 10.01 ^b^	0.185	0.030
eGFR	75.55 ± 27.33 ^a,b,c^	71.76 ± 24.24 ^c^	0.346	82.68 ± 23.16 ^a,d^	79.9 ± 23.85 ^b,d^	0.296	0.004
PNİ Score	53.50 ± 24.75	49.31 ± 12.94	0.600	55.42 ± 17.65	52.65 ± 19.83	0.292	0.105
CONUT Score	2.59 ± 2.99	2.33 ± 2.40	0.886	1.97 ± 2.05	2.07 ± 2.18	0.818	0.235

P* = comparison result of 4 groups with one-way ANOVA. a,b,c,d = the same letters in each row indicate that the difference is not significant (*p* > 0.05). **N (F/M):** number of patients (female/male); **LDH:** lactate dehydrogenase; **LDL**: low-density lipoprotein cholesterol; **HDL**: high-density lipoprotein cholesterol; **WBC:** white blood cell count; **EF:** ejection fraction; **eGFR:** estimated glomerular filtration rate; **PNI Score:** Prognostic Nutritional Index Score; **CONUT Score**: Controlling Nutritional Status Score; **MI:** myocardial infarction; **STEMI:** ST-elevation myocardial infarction; **NSTEMI**: non-ST-elevation myocardial infarction; DM: diabetes mellitus.

**Table 3:** Binary logistic regression results.

		B	SE	% 95 CI	Exp(B)	*p*
**Exitus**	Constant	−9.388	3.320		0.000	0.005
	Age	0.009	0.003	1.003–1.154	1.076	0.041
	Glucose	0.009	0.003	1.004–1.015	1.009	0.001
	Platelet	0.007	0.004	1.000–1.014	1.007	0.064
	eGFR	−0.041	0.012	0.937–0.984	0.960	0.001
	R^2^ (Cox–Snell) = 0.237 R^2^ (Nagelkerke) = 0.347 Model: X^2^(2) = 50.95. *p* < 0.001					

B: regression coefficient; SE: standard error; CI: confidence interval; Exp(B): odds ratio; *p*: significance level; eGFR: estimated glomerular filtration rate. R^2^ (Cox–Snell) and R^2^ (Nagelkerke): coefficients of determination for model fit. *p* < 0.05 was considered statistically significant.

## Data Availability

Ö.S. and M.F.U. are the guarantors of the work, and as such, had full access to all the data in the study and takes responsibility for the integrity of the data and the accuracy of the data analysis.

## Abbreviations

The following abbreviations are used in this manuscript:

ACS	Acute coronary syndrome
DM	Diabetes mellitus
STEMI	ST-elevation myocardial infarction
NSTEMI	Non-ST-elevation myocardial infarction
CVD	Cardiovascular disease
CAD	Coronary artery disease
AMI	Acute myocardial infarction
pPCI	Primary percutaneous coronary intervention
LDL	Low-density lipoprotein
HDL	High-density lipoprotein cholesterol
SPSS	Statistical Package for the Social Sciences
TG	Triglycerides
TC	Total cholesterol
PNI	Prognostic Nutrition Index
CONUT	Controlling Nutritional Status
EF	Ejection fraction
eGFR	Estimated glomerular filtration rate
